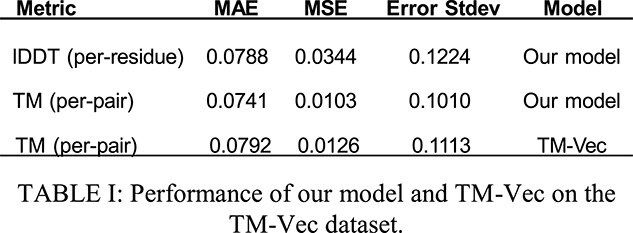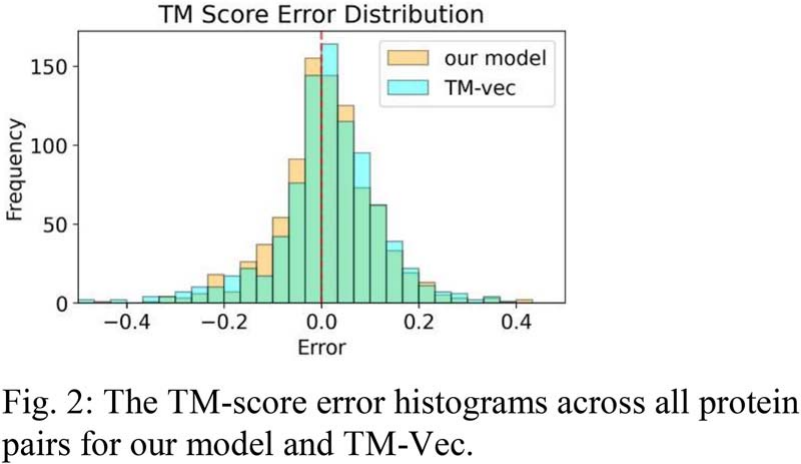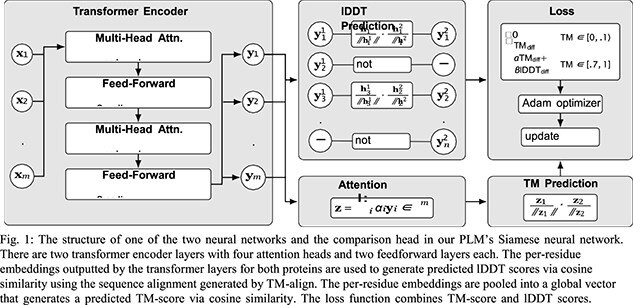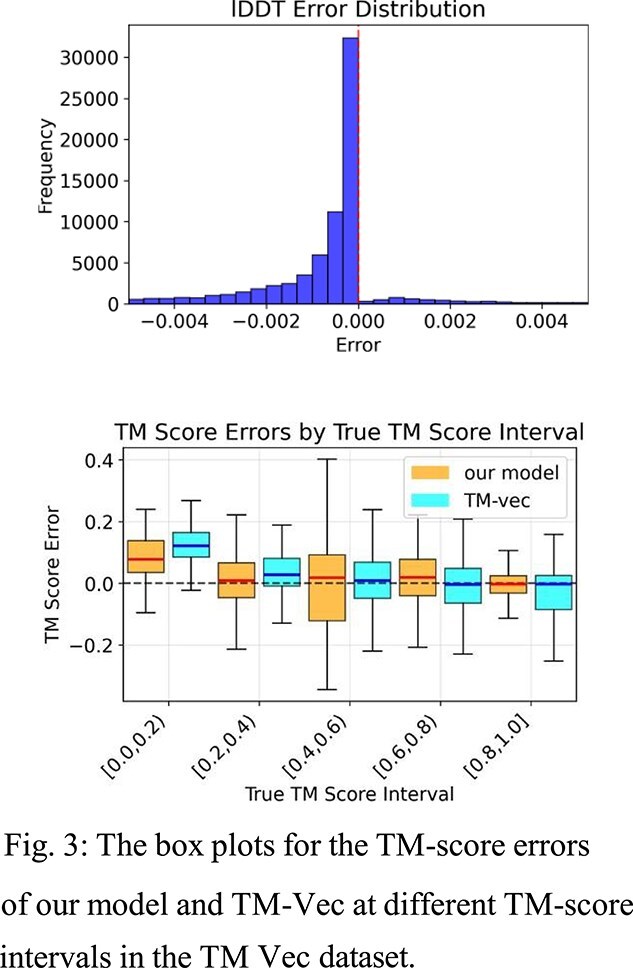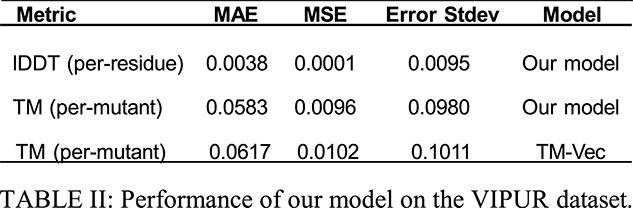# A unified protein embedding model with local and global structural sensitivity

**DOI:** 10.1093/bib/bbaf631.002

**Published:** 2025-12-12

**Authors:** Jerry Xu, Shaojun Pei, Gil Alterovitz

**Affiliations:** MIT PRIMES; Brigham and Women’s Hospital; Brigham and Women’s Hospital; Harvard Medical School

## Abstract

**Introduction:**

Many research tasks depend on identifying structural homologs of proteins, including but not limited to evolutionary analysis, peptidomimetics, and functional an- notation. However, identifying structural homologs at scale requires efficient comparison algorithms, which currently are still limited. While sequence alignment algorithms like BLAST and MMSeqs2 have become extremely optimized, they are ineffective for structural comparisons, since many structural homologs differ vastly in sequence. Meanwhile, structural alignment algorithms like TM-Align [1] and DALI.

[2] are computationally expensive due to high algorithmic complexity. Such algorithms often perform submatrix align- ment on *C_α_* distance matrices to directly superimpose proteins, which has a quadratic time complexity or worse.

In contrast, protein language models [3], or PLMs, can predict structural similarity in *O*(1) time. Utilizing the biophysi- cal prior that sequences can reconstruct structures, structural PLMs can generate sequence-based but structurally-aware em- beddings for proteins, which can then be compared via cosine similarity. Although efficient, existing structural PLMs do not recognize localized structural changes. One notable example is.

The network outputs two types of embeddings: it directly produces per-residue embeddings, and then pools them into a global embedding for the protein. Predicted TM-scores are calculated as the cosine similarity between the global em- beddings of each protein. lDDT score prediction only occurs after filtering out low TM-score protein pairs; then, using the sequence alignment generated by TM-align, we predict the lDDT scores for each pair of aligned residues as the cosine similarity of the aligned embeddings. Training occurred over 5 epochs of 300*,* 000 protein pairs.

**Results:**

We evaluated our model against two professionally curated datasets, one from the TM-Vec paper, and one from VIPUR. For the 886 proteins from the TM-Vec dataset, Table 1 evaluates our TM-score and lDDT-score prediction, as well as TM-Vec’s TM-score prediction. The TM-score error histogram is shown in Fig. 2, and the TM-score error box plot is shown in Fig. 3.

TM-Vec [4], a recently-developed PLM trained to predict TM-scores (template-modeling scores), a global similarity metric for proteins. While highly accurate for TM-scores, TM-Vec is locally unaware. We aim to create a PLM that is both globally and locally structure-aware.

**Methodology:**

Our PLM is a transformer-based Siamese neural network [5], consisting of two identical neural networks and a comparison head (see Fig. 1). Our PLM addresses both the inefficiency and local insensitivity of prior methods. We continue to generate sequence-based embeddings, resulting in efficient comparison times. Furthermore, our model utilizes a loss function that combines TM-score and a custom variation of lDDT scores (Local Distance Different Test scores) [6], which are per-residue structural similarity scores, ensuring the model captures both global and local structural features.

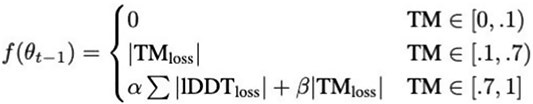

Contrastive loss was based upon the function below. *θ_t_* represents the model parameters at time *t*, which are updated based upon the loss of the previous parameters *f* (*θ*_*t −* 1_). The degree to which each type of loss (TM, lDDT) contributes to the overall loss can be adjusted via the *α, β* hyperparameters. (In our model, *α* = 0*.*7*, β* = 0*.*3.)

For the 364 proteins in the VIPUR dataset, Table 2 evaluates our TM-score and lDDT-score prediction, as well as TM-Vec’s TM-score prediction. The lDDT error histogram is shown in Fig. 4.

**Conclusion:**

Our testing results confirmed the plausibility of our framework, as our model performed similarly in TM-score prediction when compared to highly accurate models like TM- Vec while also producing accurate lDDT scores. This dual capability makes the model a potential tool in downstream research tasks, especially mutation analysis, where it could aid in the identification of deleterious mutations as well as the recognition of affected subdomains.

**References:**

1. Y. Zhang, ‘Tm-align: a protein structure alignment algorithm based on the tm-score,’ *Nucleic Acids Research*, vol. 33, no. 7, p. 2302–2309, Apr. 2005. [Online]. Available: http://dx.doi.org/10.1093/nar/gki524

2. L. Holm and C. Sander, ‘Protein structure comparison by alignment of distance matrices,’ *Journal of Molecular Biology*, vol. 233, no. 1, p. 123–138, Sep. 1993. [Online]. Available: http://dx.doi.org/10.1006/jmbi.1993.1489

3. L. Wang, X. Li, H. Zhang, J. Wang, D. Jiang, Z. Xue, and Y. Wang, ‘A comprehensive review of protein language models,’ 2025. [Online]. Available: https://arxiv.org/abs/2502.06881

4. T. Hamamsy, J. T. Morton, R. Blackwell, D. Berenberg, N. Carriero, V. Gligorijevic, C. E. M. Strauss, J. K. Leman, K. Cho, and R. Bonneau, ‘Protein remote homology detection and structural alignment using deep learning,’ *Nature Biotechnology*, vol. 42, no. 6, p. 975–985, Sep. 2023. [Online]. Available: http://dx.doi.org/10.1038/s41587-023-01917-2

5. Y. Li, C. L. P. Chen, and T. Zhang, ‘A survey on siamese network: Methodologies, applications, and opportunities,’ *IEEE Transactions on Artificial Intelligence*, vol. 3, no. 6, p. 994–1014, Dec. 2022. [Online]. Available: http://dx.doi.org/10.1109/TAI.2022.3207112

6. V. Mariani, M. Biasini, A. Barbato, and T. Schwede, ‘lddt: a local superposition-free score for comparing protein structures and models using distance difference tests,’ *Bioinformatics*, vol. 29, no. 21, p. 2722–2728, Aug. 2013. [Online]. Available: http://dx.doi.org/10.1093/bioinformatics/btt473.